# Comparative transcriptome analysis reveals key insights into male sterility in *Salvia miltiorrhiza* Bunge

**DOI:** 10.7717/peerj.11326

**Published:** 2021-04-27

**Authors:** Yan Yu, Yuanyuan Jiang, Long Wang, Yichao Wu, Jinqiu Liao, Mingzhi Zhong, Ruiwu Yang, Xingfu Chen, Qingmiao Li, Li Zhang

**Affiliations:** 1 College of Sciences, Sichuan Agricultural University, Ya‘an, Sichuan, China; 2 College of Life Science, China West Normal University, Nanchong, Sichuan, China; 3 College of Life Science, Sichuan Agricultural University, Ya‘an, Sichuan, China; 4 College of Agronomy, Sichuan Agricultural University, Wenjiang, Sichuan, China; 5 Sichuan Provincial Key Laboratory of Quality and Innovation Research of Chinese Materia Medica, Sichuan Academy of Chinese Medicine Sciences, Chengdu, Sichuan, China

**Keywords:** *Salvia miltiorrhiza* bunge, RNA-Seq, Differentially expressed genes, Male sterility

## Abstract

**Background:**

Large-scale heterosis breeding depends upon stable, inherited male sterility lines. We accidentally discovered a male sterility line (SW-S) in the F_1_progeny of a *Salvia miltiorrhiza* Bunge from Shandong, China (purple flowers) crossed with a *S. miltiorrhiza* f. alba from Sichuan, China (white flowers). We sought to provide insights into the pollen development for male sterility in* S. miltiorrhiza*.

**Methods:**

The phenotypic and cytological features of the SW-S and fertile control SW-F were observed using scanning electron microscopy and paraffin sections to identify the key stage of male sterility. Transcriptome profiles were recorded for anthers at the tetrad stage of SW-S and SW-F using Illumina RNA-Seq.

**Results:**

The paraffin sections showed that sterility mainly occurred at the tetrad stage of microspore development, during which the tapetum cells in the anther compartment completely fell off and gradually degraded in the sterile line. There was little-to-no callose deposited around the microspore cells. The tetrad microspore was shriveled and had abnormal morphology. Therefore, anthers at the tetrad stage of SW-S and fertile control SW-F were selected for comparative transcriptome analysis. In total, 266,722,270 clean reads were obtained from SW-S and SW-F, which contained 36,534 genes. There were 2,571 differentially expressed genes (DEGs) in SW-S and SW-F, of which 63.5% were downregulated. Gene Ontology (GO) enrichment analysis indicated that the differentially expressed genes were enriched in 56 functional groups (GO terms); of these, all DEGs involved in microgametogenesis and developmental maturation were downregulated in SW-S. These results were confirmed by quantitative RT-PCR. The two GO terms contained 18 DEGs, among which eight DEGs (namely: *GPAT3, RHF1A, phosphatidylinositol, PFAS, MYB96, MYB78, Cals5*, and* LAT52*) were related to gamete development. There were 10 DEGs related to development and maturation, among which three genes were directly related to pollen development (namely: *ACT3, RPK2*, and* DRP1C*). Therefore, we believe that these genes are directly or indirectly involved in the pollen abortion of SW-S. Our study provides insight into key genes related to sterility traits in *S. miltiorrhiza*, and the results can be further exploited in functional and mechanism studies.

## Introduction

Pollen development is a vital biological process in flowering plants. Abnormally developed pollen results in difficulties forming functional pollen, which leads to male sterility and can seriously affect plant reproduction. However, with the development of crop heterosis, male sterility has been more widely used in crop breeding practices, leading to improved crop yield and stress resistance. A large number of studies have shown that male sterility is mainly due to pollen abortion. Abortive initiation of the temperature-sensitive nuclear male sterile line TE5A in *Brassiana napus* occurs during the meiosis period of the pollen mother cell. Due to abnormal meiosis, dyad and tetrads could not be formed (Yan et al., 2016). The initial stage of anther abortion in chemically-induced male sterility wheat occurs from the meiosis stage to the tetrad stage. Pollen mother cells degenerate at the early stage of meiosis and are unable to further form microspores, which ultimately leads to pollen abortion (Ba et al., 2014). During anther development in *Shibataea chinensis*, the tapetum cells disassemble prematurely; the microspore mother cells and mononuclear microspores are then deformed, eventually leading to pollen abortion (Lin et al., 2016). The development of pollen is a very complex process involving the expression and regulation of many genes. Regardless of which key gene is mutated or is abnormally expressed, the result may be the abnormal development of pollen, leading to plant male sterility. An in-depth study of genes related to pollen development and their regulatory mechanisms may provide an important basis for the study of male sterility in plants and the creation of excellent sterile male lines.

*Salvia miltiorrhiza* Bunge belongs to Lamiaceae family, also known as Danshen in Chinese. Its root is important in traditional Chinese medicine (TCM). This plant has been used for thousands of years to treat various diseases, especially coronary heart disease and cerebrovascular diseases in China (Zhou, Zuo & Chow, 2005). Currently, more than 100 active ingredients have been isolated, of which tanshinone and salvianolic acid are the major compounds (Ma et al., 2015). *S. miltiorrhiza* is also an important model plant for studies of Chinese medicine due to its small number of chromosomes (2*n* = 2*x* = 16), small genome size (about 558 Mb), short life cycle, and undemanding growth requirements (Ma et al., 2012). *S. miltiorrhiza* is artificially cultivated in China and is rarely found in the wild. The current breeding practices for *S. miltiorrhiza* are based on introduction and selection, with slow progress toward genetic improvement, and a lack of excellent varieties. Heterosis breeding is an effective way to increase the yield and functional components of *S. miltiorrhiza*. However, the research on heterosis breeding of *S. miltiorrhiza* is still in its infancy, and only a few varieties have resulted from this method (Chen et al., 2016). A suitable and maintainable male sterile line is the key to heterosis breeding of *S. miltiorrhiza*. Although male sterility in *Salvia* was reported as early as 1955 (Kaul, 1988), the genetic and molecular mechanism of the male sterile line is unclear. We accidentally discovered a male sterile line, SW-S, in the F_1_ progeny of a *S. miltiorrhiza* crossed with *S. miltiorrhiza* Bge. f. *alba* C. Y. Wu et H. W. Li. We speculate that the SW-S may have cytoplasmic male sterility (CMS), with an important application value in the heterosis breeding in *S. miltiorrhiza*.

Previous transcriptome analysis of medicinal plants focused on secondary metabolite pathway exploration (Zhang et al., 2016), molecular marker mining (Wang et al., 2016), and plant stress resistance (Wei et al., 2018), ignoring their morphological development, especially the development of flower organs. The flower organs of medicinal plants are often closely related to heterosis breeding. We used RNA-Seq to investigate and compare the transcriptomes of the anthers at the tetrad stage of the male sterile plant SW-S and the control plant SW-F. The results of this study provide insights into pollen development in *S. miltiorrhiza*.

## Materials & Methods

### Plant materials

This male sterile plant was accidentally discovered in the F_1_ progeny of a *S. miltiorrhiza* (breeding lines from Shandong, China; purple flowers) crossed with *S. miltiorrhiza* f. *alba* (breeding lines from Sichuan, China; white flowers) in 2017. We temporarily named the sterile plant SW-S and the fertile plant in the F_1_ offspring SW-F. We recorded the dates of the main growth and young panicle differentiation stages in SW-S and SW-F. Overall, the anther’s developmental stages can be divided into four stages based on the microsporogenesis progression: pollen mother cells, meiotic division, tetrad, and mature pollen grains. In order to ensure the reliability of the RNA-Seq results, a randomized complete block design was used with three biological replications. Anthers with the same length (at the tetrad stage, anther length was about two mm) were mixed separately from 30 plants for each biological replication. Approximately 100 mg of material was used in RNA extraction. Anthers from the SW-S and SW-F were also harvested at the four stages for paraffin section observation. Moreover, the mature pollen grains of SW-F and SW-S were collected for observation using scanning electron microscopy (SEM).

### Morphological analysis

Photographs of the flowers were taken using a Canon 80D digital camera (Canon, Tokyo, Japan). For light microscopic observation, the anthers of SW-S and SW-F from different developmental stages were placed in the Carnoy’s fixative (methanol/acetic acid 3:1) and stored in a 4 °C refrigerator. Paraffin sections were created following the method described by Wang et al. (2015). In brief, the specimens were dehydrated using a graded ethanol series, infiltrated with xylene (Tiangen Biotech (Beijing) Co. Ltd., China) and embedded in paraffin (Sigma-Aldrich LLC., USA). 5 µm thick transverse sections were placed onto gelatin-coated glass slides (Sigma-Aldrich LLC., USA) and stained with hematoxylin (Sigma-Aldrich LLC., USA). The sections of anthers at different stages were observed using a DP70 high-resolution camera mounted on an Olympus BX51 microscope (Olympus, Tokyo, Japan). To observe pollen morphology, the mature pollen grains of SW-F and SW-S were collected, fixed in 2.5% glutaraldehyde. The fixed plant materials were then dehydrated using a graded ethanol series (50% twice, 60%, 70%, 80%, 90%, and 100% twice, 10 min per grade) and subjected to conventional critical-point drying using liquid carbon dioxide (CO_2_) as described by Karcz (2009). The material was subsequently secured to stubs before being coated with gold (Karcz, 1996). Finally, the specimens were examined using scanning electron microscopy (JEOL JSM-6360LV) at an accelerating voltage of 20 kV.

### RNA-Seq library preparation and sequencing

Total RNA from each of the six samples (three biological replications of each material) was isolated using the RNA Easy Fast Plant Tissue Kit (Tiangen Biotech (Beijing) Co. Ltd., China) according to the instructions provided by the manufacturer. For each RNA sample, the degradation and contamination were monitored using 1% RNase-free agarose gel electrophoresis. RNA integrity was assessed using the RNA Nano 6000 Assay kit of the Bioanalyzer 2100 system (Agilent Technologies, CA, USA). Messenger RNA (mRNA) was purified from total RNA using ploy-T oligo-attached magnetic beads, before breaking into short fragments (Chen et al., 2017). First-strand complementary DNA (cDNA) was synthesized using a random hexamer primer and M-MuLV Reverse Transcriptase (Roche Pharmaceutical Ltd., Switzerland). Second-strand cDNA synthesis was subsequently performed using DNA Polymerase I and M-MuLV Reverse Transcriptase. Remaining overhangs were converted into blunt ends via exonuclease/polymerase activities. After adenylation of the 3′ ends of the DNA fragments, the NEBNext Adaptor with a hairpin loop structure was ligated to prepare for hybridization (Chen et al., 2017). In order to select cDNA fragments of 250–300 bp in length, the library fragments were purified with the AMPure XP system (Beckman Coulter, Beverly, USA). Then, 3 µL of USER Enzyme (NEB, USA) was applied to size-selected, adaptor-ligated cDNA at 37 °C for 15 min, followed by 5 min at 95 °C before PCR. Then, PCR was performed using Phusion High-Fidelity DNA polymerase, Universal PCR primers, and Index (X) Primer. Next, PCR products were purified (AMPure XP system, Beckman Coulter, Beverly, USA), and their library quality was assessed on the Agilent Bioanalyzer 2100 system (Agilent Technologies Inc. USA). Lastly, Illumina sequencing was performed with the Illumina HiSeq PE150 platform by Novogene Biotech Co., Ltd. (Beijing, China).

### Data processing and differentially expressed gene (DEG) identification

Raw reads in fastq format were first processed using in-house perl scripts (Gaiarsa et al., 2019). In this step, the clean reads were obtained by removing adaptor sequences, low-quality reads (base number *Q*_phred_ ≤20 in more than 50% of the whole reads), and reads containing poly-N from raw reads. The Q20, Q30, and GC content of the clean data were calculated simultaneously. All downstream analyses were based on the clean data (Wu et al., 2017). The clean reads were then mapped to the *S. miltiorrhiza* reference genome (http://www.ndctcm.org/shujukujieshao/2015-04-23/27.html) using Hisat 2 v2.0.5 (Kim, Langmead & Salzberg, 2015). The mapped reads of each sample were assembled by StringTie (v1.3.3b) in a reference-based approach (Pertea et al., 2015). FeatureCounts v1.5.0-p3 was used to count the read numbers mapped to each gene (Liao, Smyth & Shi, 2014). Next, the fragments per kilobase million (FPKM) of each gene were calculated according to the length of the gene and read count mapped to the gene. The differentially expressed genes (DEGs) were calculated using the DESeq2 R package (1.16.1), with *q* < 0.05, —log_2_ fold change— ≥2 (Wang et al., 2013).

### Functional analysis of DEGs

We annotated the DEGs on the basis of a set of sequential Basic Local Alignment Search Tool (BLAST) iterations, designed to find the most descriptive annotation for each sequence. The DEGs were compared with sequences in the National Center for Biotechnology Information’s (NCBI) nonredundant (Nr) protein and nucleotide (Nt) databases (http://www.ncbi.nlm.nih.gov/). Gene Ontology (GO) enrichment analysis was performed using agriGO (http://systemsbiology.cau.edu.cn/agriGOv2/index.php) (Du et al., 2010). We detected which of the DEGs were significantly enriched in GO terms using a *p* < 0.05 as the threshold of significance.

### Quantitative RT-PCR (qRT-PCR) validation

First-strand cDNA synthesis was conducted using 5 µg of total RNA from the anthers of SW-S and SW-F at the tetrad stage with a PrimeScript RT reagent kit (Takara, Dalian, China) according to the manufacturer’s instructions. The *SmActin* gene (DQ243702) was employed as an internal control for qRT-PCR (Yang et al., 2010). The primers for the selected genes and *SmActin* gene were designed using Beacon Designer 7, and are listed in Table S1. qRT-PCR assays were performed with SYBR Green Dye (Takara, Dalian, China) using a Bio-Rad CFX96 real-time PCR platform (Bio-Rad, Hercules, CA, USA) according to the following program: 95 °C for 5 min, followed by 40 cycles at 95 °C for 5 s and 55−63 °C (annealing temperature varied according to primers) for 30 s. All assays for a particular gene were performed three times synchronously under identical conditions, and RNA transcript fold changes were calculated using the 2^−ΔΔ*Ct*^ method (Livak & Schmittgen, 2001).

## Results

### Phenotypic characteristics of SW-S and SW-F anthers

**Figure 1:**
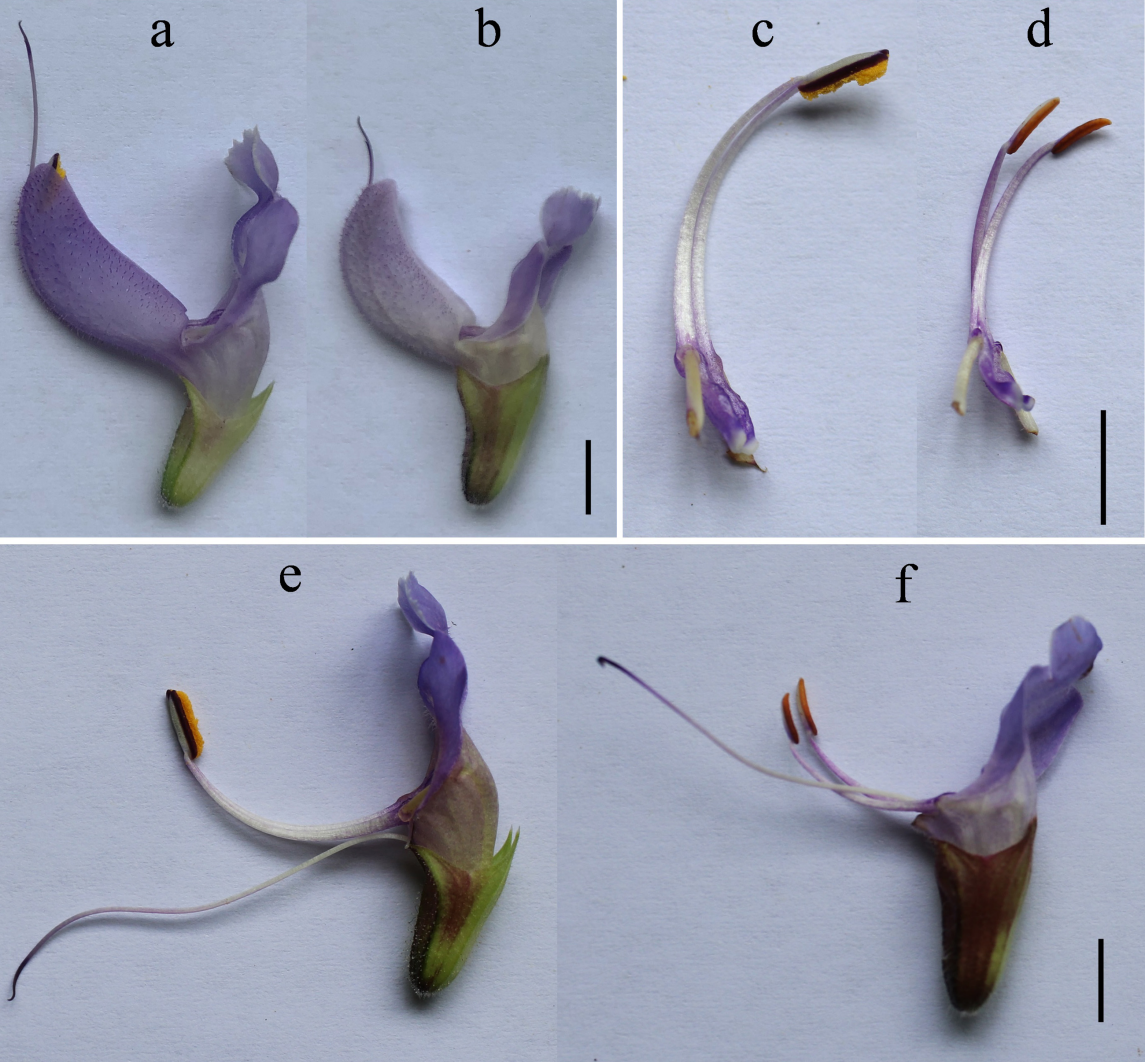
Morphology of the fertility line SW-F (A, C, E) and sterile line SW-S (B, D, F). (A, B) The corolla of SW-F and SW-S. (C, D) Anther and filament morphology of SW-F and SW-S. (E, F) Stamen and stigma morphology of SW-F and SW-S. Scale bars represent five mm.

The male sterile line SW-S was discovered in the F_1_ progeny of an *S. miltiorrhiza* (from Shandong, China) crossed with *S. miltiorrhiza* f. *alba* (white flowers). Twenty-one individuals of 40 F_1_ progenies in *S. miltiorrhiza* × *S. miltiorrhiza* f. *alba* showed male sterility, and the remaining 19 individuals were fertile (almost 1:1). However, crossing *S. miltiorrhiza* f. *alba* as the mother and *S. miltiorrhiza* as the father, produced F_1_ offspring that were all fertile. So, SW-S may belong to the CMS. To confirm the stability of sterility, the roots of sterile plants were used for asexual reproduction for 2 consecutive years (2018 and 2019), and the plants with asexual reproduction were planted in the experimental field of China West Normal University, Nanchong, Sichuan, China (30°49′ north (N), 106°3′ east (E)). The development of anthers in the sterile plants was observed in April of the following year. The results showed that the anthers of all sterile plants were abnormal and could not produce normal pollen, suggesting that the sterility was stable. The comparison of agronomic traits showed that there were no significant differences in overall morphology and growth except for the floral organs of the sterile line SW-S and its fertility control SW-F. The corolla of the fertility control SW-F was blue-purple, while the flower color of the sterile line SW-S was shallow (Figs. 1A, 1B); in addition, all parts of the flower organs in SW-S were smaller than those in the fertile plants (Figs. 1E–1F). The day that SW-F flowered, the anther’s normal dehiscing was accompanied by the release of a large number of mature pollen grains. By contrast, the SW-S anthers did not dehisce or had a very small amount of pollen, which was found to be inactive (Figs. 1C, 1D).

**Figure 2:**
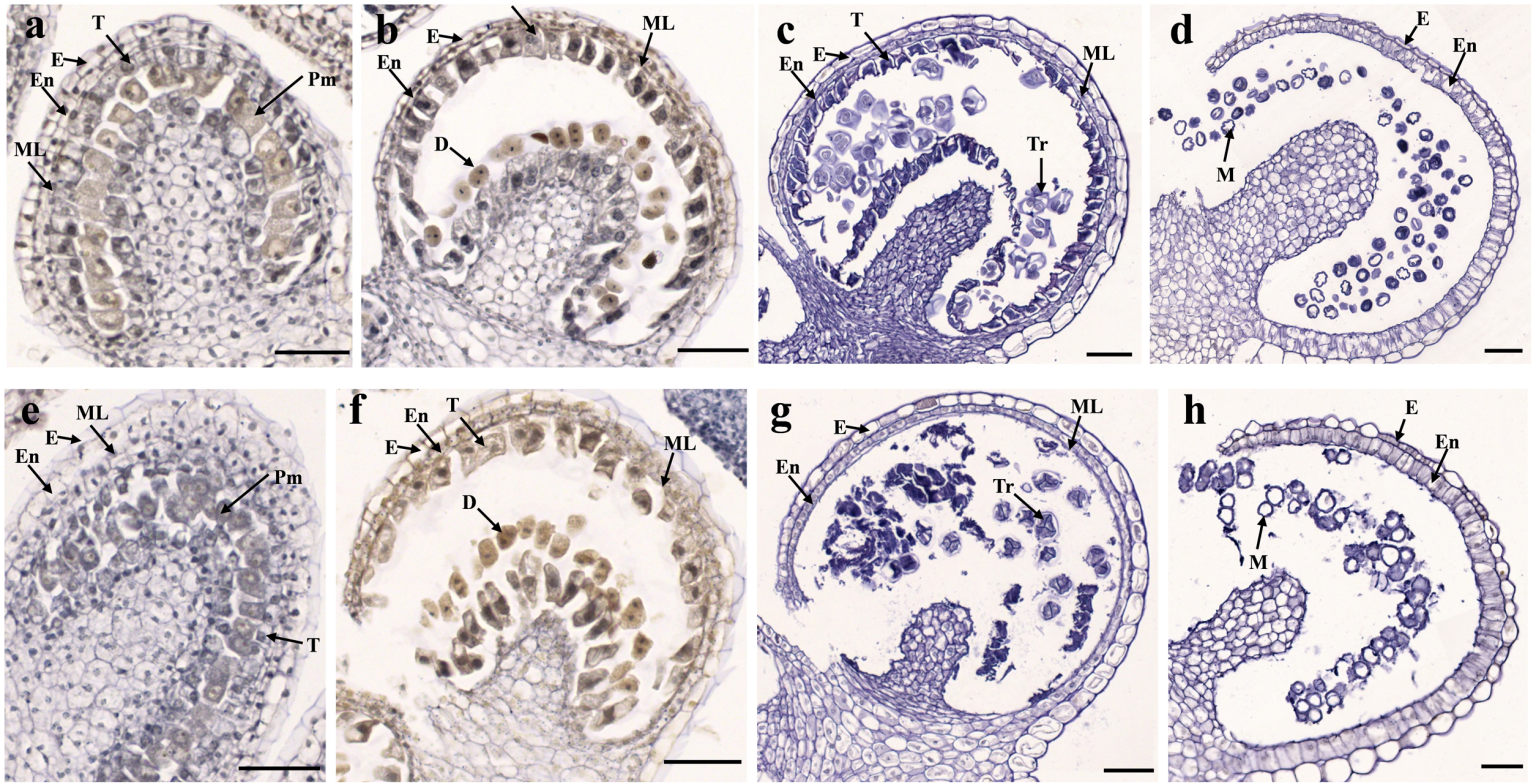
Transverse sections of anthers from fertile line SW-F (A–D) and sterile line SW-S (E–H) of *S. miltiorrhiza* plants during different maturation stages. (A, E) Pollen mother cells stage of SW-F and SW-S. (B, F) Meiotic division stage of SW-F and SW-S. (C, G) Tetrad stage of SW-F and SW-S. (D, H) Mature pollen grains stage of SW-F and SW-S. Scale bars represent 50 µm. E, epidermis; En, endothecium; ML, middle layer; T, tapetum; Pm, pollen mother cells; D, dyad; Tr, tetrads; M, microspore.

**Figure 3:**
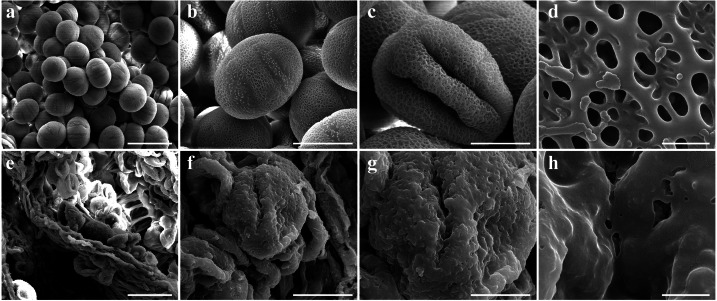
Comparison of scanning electron microscope (SEM) images of SW-F (A–D) and SW-S (E–H) pollen grains. Scale bars represent 50 µm in A and E, 20 µm in B, C, F and G, 1 µm in D and H.

To determine the abortive morphological features of SW-S, we collected SW-S and SW-F anthers at four development stages: pollen mother cells, meiotic division, tetrad, and mature pollen grains. In order to accurately observe the cytological structure of the sterile anthers, we prepared paraffin sections of anthers from the first three stages. As shown in Fig. 2, there were clear differences between the fertile anthers and sterile anthers. The normal anther structure involved the epidermis, endothecium, middle layer, and tapetum from outside to inside. Compared with the SW-F, the sterile line SW-S at the pollen mother cell stage had an unclear cell arrangement layer, while the tapetum cells were irregular sharped (Figs. 2A, 2E). During meiosis, the tapetum cells near the pollen sac began to detach in the sterile line (Figs. 2B, 2F). After entering the tetrad stage, all tapetum cells in the pollen sac completely fell off and gradually degraded in the sterile line. Furthermore, there was little or no callose deposited around microspore cells. The tetrad microspore was shriveled and had an abnormal morphology (Figs. 2C, 2G). Hence, we considered the tetrad period to be the critical period for pollen abortion. With the degradation of the callose, microspores were released. The microspores of normal fertile plants were fusiform or subglobose, with six germ furrows (Fig. 2D). However, the microspores of the sterile line were fused together, irregular in shape, and uneven in size, with no obvious germ furrows (Fig. 2H). The results of SEM showed that the normal pollen grains were long and spherical, while the germ furrows were distinct (Fig. 3). In addition, the exine of normal pollen had clear ornamentation, a smooth murus, and obvious oval lumina (Fig. 3). Compared with normal pollen grains, sterile plants had withered pollen, which was sticky and could not be dispersed (Fig. 3). The surface of the sterile pollen had few germ furrows, the surface ornamentation was not clear, and there were no distinct lumina (Fig. 3).

### Transcriptome sequencing

To obtain key insights into male sterility for *S. miltiorrhiza* at the transcriptional level, we employed the Illunina HiSeq PE150 platform for transcriptome sequencing analysis using the anthers from the sterile line SW-S and the fertile line SW-F at the tetrad stage. Anthers were analyzed three times with a total of three biological replicates, and the sequencing read lengths were 150 bp. After filtering out adaptor sequences, low-quality reads, and reads containing ploy-N from raw reads, 266,722,270 clean reads were obtained, with 130,305,496 reads from the sterile line and 136,416,774 from the fertile line. The average GC content was 47.2%, and the Q20 percentage exceeded 97%. The clean reads obtained from SW-S and SW-F were matched with the *S. miltiorrhiza* reference sequence, where the mapping rates ranged from 88.19% to 89.65% (Table 1). The productivity and quality of the sequencing indicated that the RNA-seq data were sufficient for further analysis.

### Identification of differentially expressed genes

**Figure 4:**
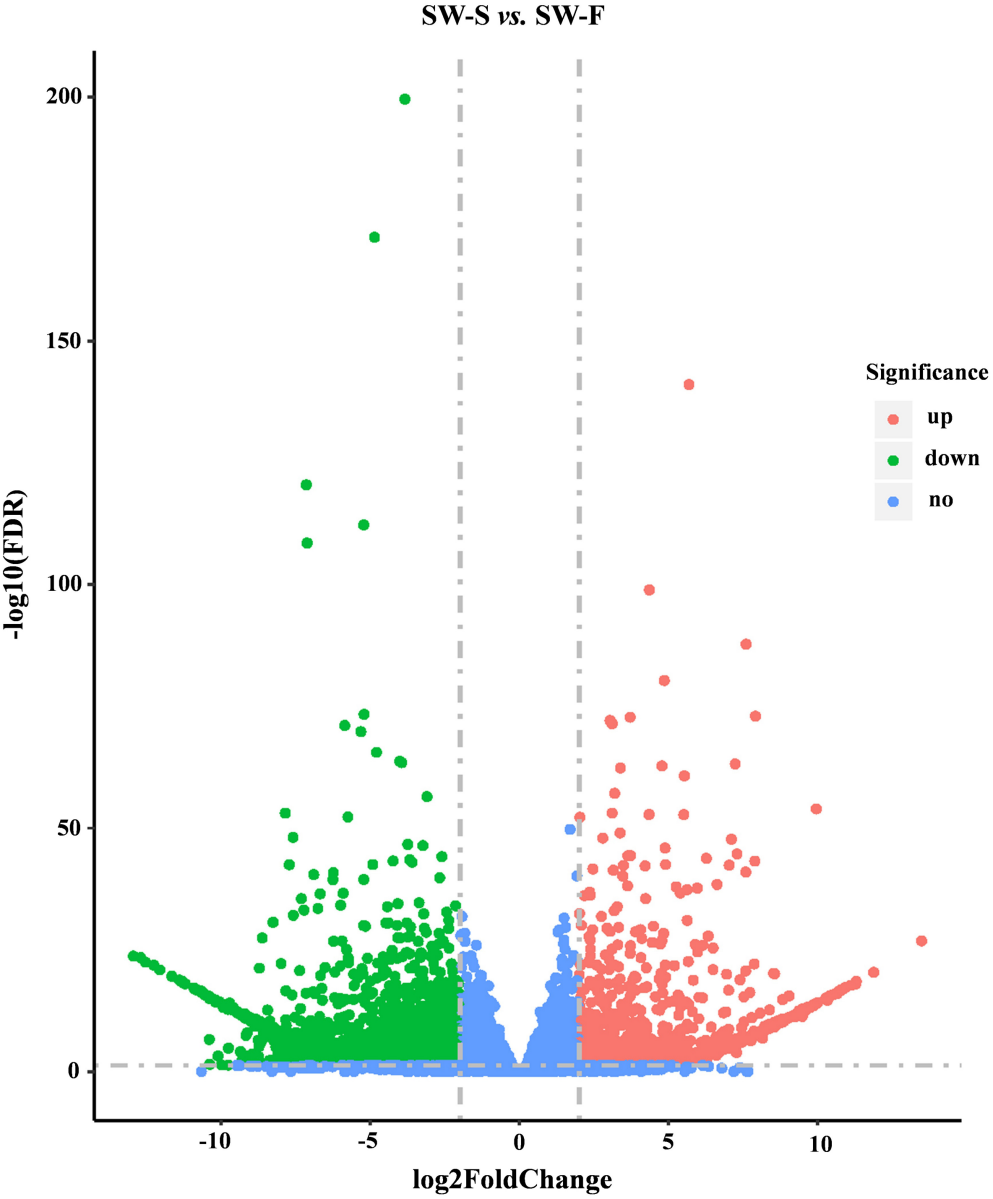
Volcano plots showing DEGs between SW-S and SW-F during tetrad stage. Blue dots indicate no significant difference. Red dots indicate significantly upregulated genes, a total of 939 genes. Green dots indicated significantly downregulated genes, a total of 1,632 genes. FDR, false discovery rate.

A total of 36,534 genes were detected from SW-S and SW-F, using RNA-Seq, of which 4,607 were novel genes (unannotated genes compared to the reference genome). To make it easier to distinguish these novel genes, we added “novel” before the gene identifier (ID). To determine significant differences in gene expression levels in SW-S and SW-F, we used the following thresholds: *q* <0.05, and —log_2_ fold change— ≥2. In total, 2,571 genes were differentially expressed in SW-S and SW-F anthers (Fig. 4). These DEGs were comprised of 939 upregulated genes and 1,632 downregulated genes in the SW-S anthers compared with SW-F anthers (Table S2). Among these DEGs, 2,152 genes could be mapped to the reference gene, whereas 419 novel genes could not be mapped to the reference gene.

**Table 1:** Summary and evaluation of sequencing results.

Sample	Raw reads	Clean reads	Clean bases	Error rate (%)	Q20	Q30	GC (%)	Total mapping ratio (%)
SW-S-1	46439240	44313566	6.65G	0.03	97.94	93.93	47.02	88.89%
SW-S-2	45508726	43202992	6.48G	0.03	97.84	93.69	47.39	89.65%
SW-S-3	46835412	42788938	6.42G	0.02	98.14	94.46	47.26	89.51%
SW-F-1	48591472	45881856	6.88G	0.03	97.52	92.79	47.15	88.19%
SW-F-2	47759126	45590064	6.84G	0.03	97.73	93.44	47.28	88.71%
SW-F-3	47421082	44944854	6.74G	0.03	97.85	93.73	47.08	88.95%

### GO enrichment analysis of DEGs

**Figure 5:**
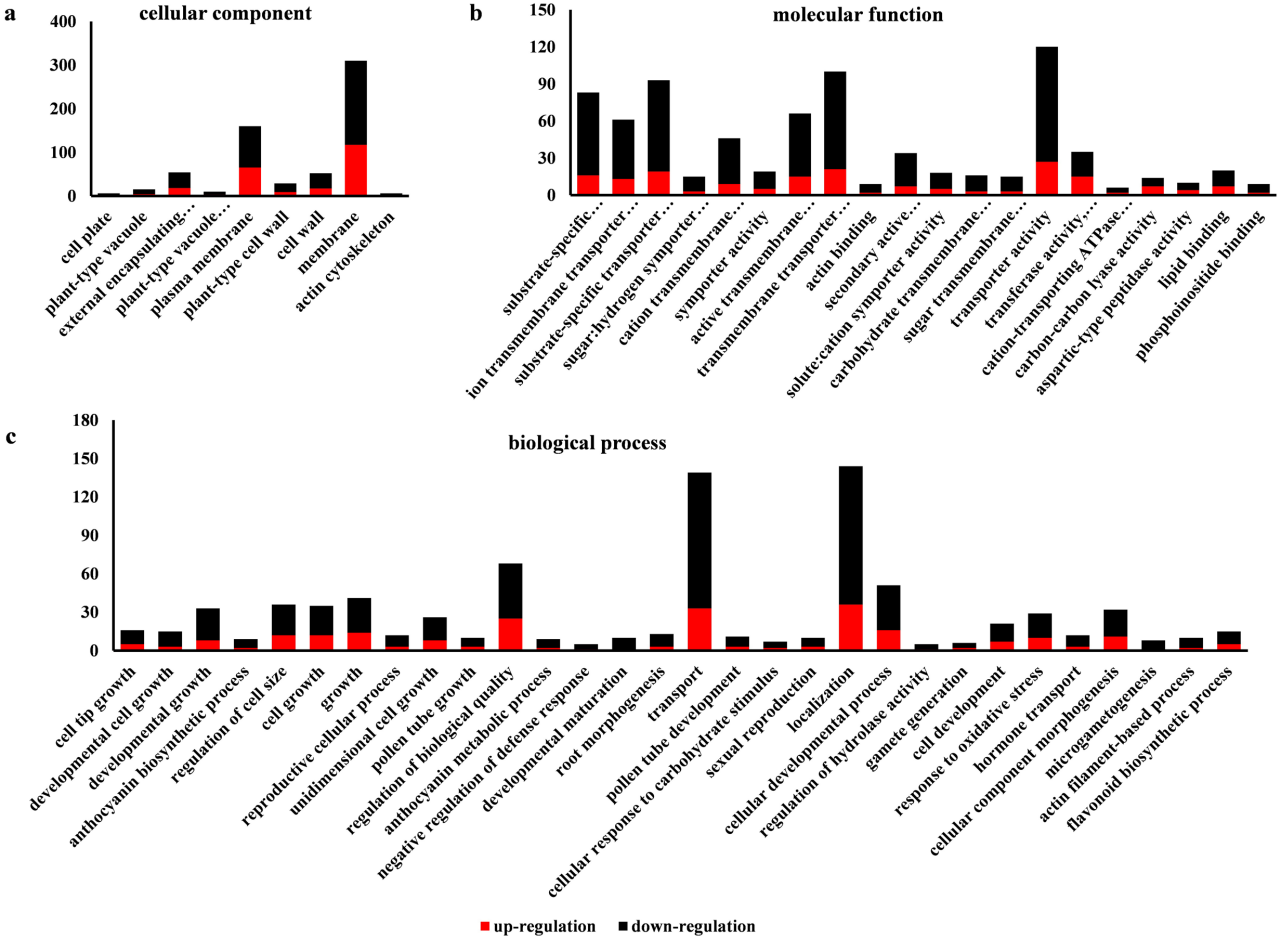
Gene Ontolgoy (GO) classifications of differentially expressed genes (DEGs) in SW-S and SW-F during the tetrad stage. (A) Cellular component, (B) molecular function, (C) biological process.

Go is a database established by the Gene Ontology Consortium. It aims to establish a semantic vocabulary standard for defining and describing the functions of genes and proteins applicable to various species, which can be updated with the continuous development of research (Ye et al., 2006). After enrichment analysis, the DEGs found in SW-S and SW-F were annotated according to 56 functional groups (Fig. 5). Among the biological processes, the DEGs were mainly associated with the transport, localization, and regulation of biological quality. In terms of cellular components, the DEGs were mainly related to the membrane and plasma membrane. In addition, transporter activity, transmembrane transporter activity, substrate-specific transporter activity, substrate-specific transmembrane transporter activity, active transmembrane transporter activity, and ion transmembrane transporter activity constituted the related molecular functions. There were more downregulated DEGs in SW-S than upregulated DEGs for most GO terms (Fig. 5). The genes involved in microgametogenesis and developmental maturation were all downregulated in SW-S (Table 2). Among the top 20 enriched GO terms, only cell tip growth was considered to be significantly enriched *(p* <0.001) (Table S3). The DEGs found in different GO terms may provide valuable insight for studying anthers development in SW-S.

### Validation of key DEGs by quantitative RT-PCR (qRT-PCR)

**Figure 6:**
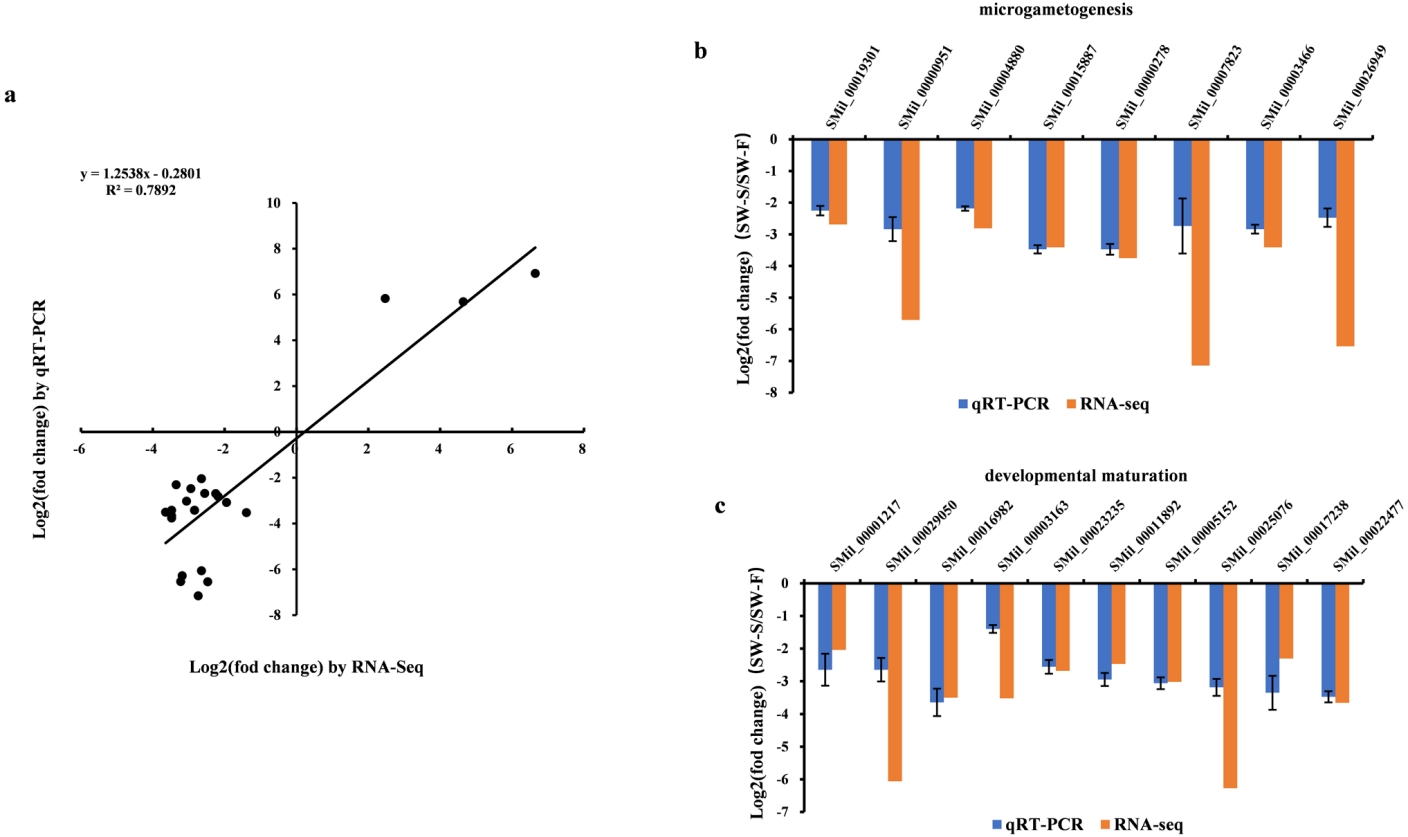
qRT-PCR validation of the RNA-Seq results for some differentially expressed genes (DEGs). Log2(fold change) represents the logarithm of the fold change in expression for SW-S relative to SW-F. (A) Comparison of expression levels measured by RNA-Seq and qRT-PCR for selected 23 DEGs. (B) DEGs related to microgametogenesis. (C) DEGs related to developmental maturation.

qRT-PCR was performed on 23 DEGs, including five random DEGs (three upregulated genes and two downregulated genes), eight DEGs related to microgametogenesis, and 10 DEGs involved in developmental maturation. We compared the results obtained from qRT-PCR with those generated from the RNA-Seq analysis of the transcripts. For these DEGs, the expression trends obtained using the two methods were consistent, with a correlation coefficient of *R*^2^ = 0.7892 (Fig. 6A). Although quantitative differences were found between the two methods to some extent, the trends were consistent. Differences in the level of expression of these genes were also reasonable because of the different methods employed. The results of qRT-PCR analysis further confirmed that eight genes related to microgametogenesis and 10 genes related to developmental maturation were all downregulated in SW-S (Figs. 6B, 6C). These genes may be related to male sterility for *S. miltiorrhiza*.

**Table 2:** The DEG involved in microgametogenesis and developmental maturation.

GO accession	GO term	Gene ID	log2FoldChange (SW-S/SW-F)	BLAST matching accession No.	Gene description
GO:0055046	microgametogenesis	SMil_00019301	−2.69	XM_025960379.1	glycerol-3-phosphate acyltransferase 3 [*Panicum hallii*]
GO:0055046	microgametogenesis	SMil_00000951	−5.71	XP_011090770.1	E3 ubiquitin-protein ligase RHF1A isoform X2 [*Panicum hallii*]
GO:0055046	microgametogenesis	SMil_00004880	−2.81	XP_011100557.1	phosphatidylinositol 3-kinase, root isoform isoform X1 [*Sesamum indicum*]
GO:0055046	microgametogenesis	SMil_00015887	−3.41	TEY34894.1	phosphoribosylformylglycinamidine synthase [*Salvia splendens*]
GO:0055046	microgametogenesis	SMil_00000278	−3.75	KF059450	MYB96 [*Salvia miltiorrhiza*]
GO:0055046	microgametogenesis	SMil_00007823	−7.15	KF059432	MYB78 [*Salvia miltiorrhiza*]
GO:0055046	microgametogenesis	SMil_00003466	−3.41	TEY89288.1	callose synthase 5 [*Salvia splendens*]
GO:0055047	microgametogenesis	SMil_00026949	−6.54	P13447.1	Anther-specific protein LAT52 [*Erythranthe guttatus*]
GO:0021700	developmental maturation	SMil_00001217	−2.04	XP_011081924.1	potassium channel AKT1 [*Sesamum indicum*]
GO:0021700	developmental maturation	SMil_00029050	−6.06	KX591571.1	actin 3 (ACT3) [*Erythranthe lewisii*]
GO:0021700	developmental maturation	SMil_00016982	−3.5	ABR92336.1	putative translationally controlled tumor protein [*Salvia miltiorrhiza*]
GO:0021700	developmental maturation	SMil_00003163	−3.52	PIN15638.1	translationally controlled tumor protein [*Handroanthus impetiginosus*]
GO:0021700	developmental maturation	SMil_00023235	−2.68	XP_012847032.1	potassium channel AKT1-like [*Erythranthe guttata*]
GO:0021700	developmental maturation	SMil_00011892	−2.47	XM_012996202.1	LRR receptor-like serine/threonine-protein kinase RPK2 [*Erythranthe guttatus*]
GO:0021700	developmental maturation	SMil_00005152	−3.02	XM_011079749.2	myosin-17-like [*Sesamum indicum*]
GO:0021700	developmental maturation	SMil_00025076	−6.27	XM_011092661.2	xyloglucan 6-xylosyltransferase 2 [*Sesamum indicum*]
GO:0021700	developmental maturation	SMil_00017238	−2.3	XM_012990647.1	dynamin-related protein 1C [*Erythranthe guttatus*]
GO:0021700	developmental maturation	SMil_00022477	−3.66	XM_019327291.1	type I inositol polyphosphate 5-phosphatase 5-like [*Ipomoea nil*]

## Discussion

*S. miltiorrhiza* is widely used in traditional Chinese medicine. It has multiple clinical and pharmacological properties and is used in the treatment of cardiovascular and cerebrovascular diseases, as well as hyperlipidemia (Zhou, Zuo & Chow, 2005). However, compared with other model plants such as *Arabidopsis thaliana* and rice, there is little basic molecular research into *S. miltiorrhiza* and its genetic background is poorly established. In recent years, transcriptome sequencing using the Illumina platform has been employed as a powerful tool with a wide range of applications, such as cell-type-specific biological processes, to address fundamental problems related to biology on an evolutionary time scale (Liu et al., 2020). The molecular mechanisms related to secondary metabolism, especially the synthesis of tanshinone and salvianolic acid, have been relatively well-studied in *S. miltiorrhiza*, while the molecular and genetic mechanisms related to morphological development are not well-studied (Zhan et al., 2019; Yang et al., 2013). This is mainly due to the low number of mutants currently found in *S. miltiorrhiza*. We identified a male sterile line SW-S in the F_1_ progeny of an *S. miltiorrhiza* (purple flowers) crossed with *S. miltiorrhiza* f. *alba* (white flowers). The results of paraffin sectioning showed that there was little or no callose deposited around microspore cells at the tetrad stage of stamen development, which may be the main cause of male sterility. This line is not only of great significance for studying the flower development of *S. miltiorrhiza*, but it can also provide parent material for hybrid breeding. In order to elucidate the molecular mechanism of male sterility, we performed RNA-Seq analysis of anthers at the tetrad stage for the sterile line SW-S and fertile control SW-F.

### Male sterility may occur at the stage of microgametogenesis

Illumina sequencing of SW-S and SW-F produced 130,305,496 and 136,416,774 clean reads, respectively, with a total of 36,534 genes. Compared with SW-F, 2,571 DEGs were detected, and 63.5% of these genes were downregulated. GO enrichment analysis of these DEGs showed that two GO terms, microgametogenesis (GO:0055046) and developmental maturity (GO:0021700), may be related to the male sterility of *S. miltiorrhiza*, as genes related to these two GO terms were all downregulated. The results of qRT-PCR also confirmed that, compared with SW-F, the expression of these genes in SW-S was significantly reduced. We attempted Kyoto Encyclopedia of Genes and Genomes (KEGG) pathway enrichment analysis on 2,571 DEGs; however, approximately 400 DEGs were enriched in terms of KEGG pathways.

Pollen is produced in the anther during two successive developmental phases, microsporogenesis and microgametophyte development. Microsporogenesis includes a series of progressive developmental stages from sporogenous cells to haploid unicellular microspores. During microsporogenesis, the diploid sporogenous cells differentiate into microspore mother cells which undergo meiotic division to form four haploid microspores (tetrad) enclosed in the callose envelope. Microgametogenesis consists mainly of the process of releasing haploid microspore from the callose envelop and producing mature male gametophytes (Tütüncü, 2014). The results of paraffin sections showed that the meiosis of SW-S pollen mother cells was normal, and a normal tetrad could be produced. Therefore, we inferred that male sterility likely occurs at the stage of microgametogenesis. The RNA-Seq results also further confirmed that all DEGs related to microgametogenesis and developmental maturity were downregulated.

### DEGs involved in microgametogenesis

There were eight DEGs associated with microgametogenesis, including *glycerol-3-phosphate acyltransferase 3* (*GPAT3*), *E3 ubiquitin-protein ligase RHF1A*, *phosphatidylinositol 3-kinase*, *phosphoribosylformylglycinamidine synthase* (*PFAS*), *MYB96*, *MYB78*, *callose synthase 5*, and *anther-specific protein LAT52*. These genes play an important role in the development of pollen. For example, a transfer (T)-DNA insertional mutant in the *glycerol-3-phosphate acyltransferase* (*GPAT*) gene exhibits abnormal tapetum morphology and defects in the formation of the pollen wall (Zheng et al., 2003). Studies have shown that the *E3 ubiquitin-protein ligase RHF1* gene plays an important role in gametogenesis. Double mutants of *rhf1a/rhf2a* in *Arabidopsis* lead to defects in the formation of male gametophytes due to interphase retardation of the mitotic cell cycle during the microspore stage of pollen development (Liu et al., 2008). Phosphatidylinositol 3-kinase is essential for vacuolar reorganization and nuclear division during pollen development in *Arabidopsis* (Lee et al., 2008). Phosphoribosylformylglycinamidine synthase (PFAS) is an essential enzyme in the de novo synthesis of purine. It has been reported that a missense mutation in PFAS can lead to the death of embryo in cattle (Michot et al., 2017). However, there is no systematic report of the role of PFAS in pollen development in plants. We observed that the *PFAS* gene was downregulated in the anthers of sterile line SW-S. Therefore, we speculate that the *PFAS* gene may be related to male sterility of plants. As the largest family of transcription factors in plants, *MYB* s play an important regulatory role in growth and development. A large number of studies have shown that R2R3-type *MYB* transcription factors play an important role in the regulatory pathways of anther development and pollen formation, including tapetum development, callose deposition and degradation, transportation of photosynthetic products, anther cracking, and male gametophyte formation (Higginson, Li & Parish, 2010; Xin et al., 2016). *MYB96* and *MYB78* are R2R3-type transcription factors; thus, the downregulation of these two genes may be related to the male sterility of *S. miltiorrhiza*. The *anther-specific protein LAT52* gene is related to late pollen development. The RNA of *LAT52* appears after the mitosis of the microspore, with its expression gradually increasing with microspore development, reaching its peak in mature pollen (Twell et al., 1989). Therefore, *LAT52* may be related to pollen maturation.

Among the eight DEGs related to microgametogenesis, we believe that the *callose synthase 5* gene (Cals5) is the most closely related to the male sterility of *S. miltiorrhiza*. Callose, primarily composed of *β*-1,3 glucan, separates developing pollen grains, preventing their exine walls from fusing (Nishikawa et al., 2005). *Cals5* encodes a *β*-1,3 glucan synthase required for exine formation during microgametogenesis and for pollen viability in *Arabidopsis* (Dong et al., 2005). Mutations in the *Cals5* gene disrupt callose formation around developing microspores, causing aberrations in exine patterning, degeneration of developing microspores, and pollen sterility (Verma & Hong, 2001). We found that the *Cals5* gene was downregulated in the male sterile line SW-S. Observation of paraffin sections also revealed little or no callose around the tetraploid gametes of SW-S. Therefore, we speculate that downregulation of the *Cals5* gene results in a decrease in callose around the developing pollen, leading to male sterility.

### DEGs related to developmental maturity

There were 10 DEGs associated with developmental maturity, including *potassium channel AKT1* (containing two homologous genes), *actin 3* (*ACT3*), *putative translationally controlled tumor protein* (*TCTP*) (containing two homologous genes), *LRR receptor-like serine/threonine-protein kinase RPK2*, *myosin-17-like, xyloglucan 6-xylosyltransferase 2*, *dynamin-related protein 1C (DRP1C)*, and *type I inositol polyphosphate 5-phosphatase 5-like*. Among these DEGs, three genes were reported to be related to pollen maturation: *ACT3*, *RPK2*, and *DRP1C*. In *Arabidopsis*, expression of the *ACT3* gene in mature pollen is higher than that in other tissues (An et al., 1996). This may indicate that the *ACT3* gene is related to pollen maturation. *RPK2* is a key regulator of anther development in *Arabidopsis*. Two *RPK2* T-DNA insertional mutants (rpk2-1 and rpk2-2) displayed enhanced shoot growth and male sterility due to defects in anther dehiscence and pollen maturation (Mizuno et al., 2010). The dynamin-related protein (DRP) family is essential for gametophyte development in plants. Backues et al. (2010) demonstrated that both male and female gametes require at least one functioning DRP family member (DRP1 and DRP2) to progress beyond the single nucleate stage of development. Previous studies found that *drp1C-1* mutants exhibited male gametophytic lethality in *Arabidopsis*, displaying small and shriveled pollen grains that do not germinate (Kang, Rancour & Bednarek, 2003). There were no other genes found to be directly related to pollen development, however, other genes may indirectly participate in the regulation of pollen maturity and requires further in-depth study.

## Conclusions

In order to isolate and identify genes associated with male sterility in *S. miltiorrhiza*, we used RNA-Seq to investigate and compare the transcriptomes of anthers at the tetrad stage from the male sterile plant SW-S and control plant SW-F. The results showed that 266,722,270 clean reads were obtained from SW-S and SW-F, which contained 36,534 genes. There were 2,571 differentially expressed genes (DEGs) in SW-S and SW-F, of which 63.5% were downregulated. GO enrichment analysis showed that the differentially expressed genes were enriched in 56 functional groups (GO terms), among which the DEGs involved in microgametogenesis and developmental maturation were all downregulated in SW-S. These results were confirmed using quantitative RT-PCR. These two GO terms contained 18 DEGs, among which eight DEGs (*GPAT3, RHF1A, phosphatidylinositol, PFAS, MYB96, MYB78, Cals5,* and *LAT52*) were related to gamete development. There were 10 DEGs related to development and maturation, among which three genes were directly related to pollen development: *ACT3*, *RPK2*, and *DRP1C*. Therefore, we believe that these genes are directly or indirectly involved in the pollen abortion of SW-S. Our study provides comprehensive insight into the key genes related to sterility in *S. miltiorrhiza*, and the results can be further explored in functional and mechanism studies.

##  Supplemental Information

10.7717/peerj.11326/supp-1 Supplemental Information 1 Primers used for gene expression analysis for qRT-PCR

10.7717/peerj.11326/supp-2 Supplemental Information 2 The differentially expressed genes between SW-S vs SW-F

10.7717/peerj.11326/supp-3 Supplemental Information 3 The top enriched 20 GO terms.

10.7717/peerj.11326/supp-4 Supplemental Information 4 Raw numerical data for Fig. 6A. Comparison of expression levels measured by RNA-Seq and qRT-PCR for selected 23 DEGs.

10.7717/peerj.11326/supp-5 Supplemental Information 5 Raw numerical data for Figs. 6B and 6C. DEGs related to microgametogenesis and developmental maturation.

## Additional Information and Declarations

### Data Availability

The following information was supplied regarding data availability: The sequences are available at the NCBI Sequence Read Archive (SRA): PRJNA682684.
